# Isolated Flexor Hallucis Longus Tendon Transfer for Chronic Achilles Tendon Rupture: Systematic Review and Meta-Analysis

**DOI:** 10.3390/healthcare13212751

**Published:** 2025-10-30

**Authors:** Yasmine J. Khair, Hugh Milchem, Maamoun Adra, Anthony Fong, Thant Htoo Nyan, Shayndhan Sivanathan, Hayato Nakanishi, Christian A. Than, Nadim Tarazi, Constantinos Loizou, Marcus Mumme

**Affiliations:** 1 School of Medicine, University of Nicosia, 2417 Nicosia, Cyprus; khair.yas@live.unic.ac.cy (Y.J.K.);; 2 Cambridge University Hospitals NHS Foundation Trust, Cambridgeshire CB2 0QQ, UK; 3 School of Health & Medical Sciences, St George’s University of London, London SW17 0RE, UK; 4 Mid and South Essex NHS Foundation Trust, Westcliff-on-Sea SS0 0RY, UK; 5 Oxford University Hospitals NHS Foundation Trust, John Radcliffe Hospital, Headley Way, Headington, Oxford OX3 9DU, UK; 6 Department of Surgery, Mayo Clinic, Rochester, MN 55905, USA; 7 School of Biomedical Sciences, The University of Queensland, St Lucia, Brisbane 4072, Australia; 8 Oxford University Hospitals NHS Foundation Trust, Nuffield Orthopaedic Centre, Windmill Rd, Oxford OX3 7LD, UK; 9 SportClinic Zürich, Klinik Hirslanden, 8032 Zürich, Switzerland; 10 Department of Biomedicine, Tissue Engineering Laboratory, University of Basel, 4031 Basel, Switzerland

**Keywords:** Achilles repair, autograft, calcaneal rupture, repair, tendon injuries, tendon transfer

## Abstract

Background: Chronic Achilles tendon ruptures (CATRs) pose a clinical challenge because guidelines on optimal treatment modalities are lacking. This meta-analysis aims to investigate the use of Flexor Hallucis Longus (FHL) tendon transfer as a treatment option. Methods: A literature search was performed across multiple databases from inception until 31 July 2025. The databases searched included Ovid MEDLINE^®^, EMBASE (Elsevier), Cochrane Central Register of Controlled Trials and Cochrane Database of Systematic Reviews. Included studies presented CATR patients of all ages, with no previous surgeries to the ankle, who were managed with an FHL tendon transfer (PROSPERO ID: CRD42023489724). Results: Sixteen studies met the eligibility criteria with 323 patients. For functionality, baseline AOFAS-AH (American Orthopaedic Foot & Ankle Society—Ankle Hindfoot) scores were 56.85 (95% CI: 51.03–62.68, I^2^ = 96%). At ≥12 months post-operative follow-up, AOFAS-AH scores were 91.46 (95% CI: 88.45, 94.48, I^2^ = 93%). ATRS (Achilles Tendon Rupture Score) at baseline was reported as 31.04 (95% CI: 5.80, 56.28, I^2^ = 99%). At ≥12 months post-operative follow-up, ATRS amounted to 90.73 (95% CI: 83.69, 97.77, I^2^ = 89%). Overall complication rates were 7.5% (CI: 0.04,0.11, I^2^ = 40%), consisting of superficial infections at 4.2% (95% CI: 0.01, 0.07, I^2^ = 0%), activity limitations at 4% (95% CI: 0.01, 0.08, I^2^ = 0%) and disturbed wound healing at 3.8% (95% CI: 0.01, 0.06, I^2^ = 0%). The minimum clinically important difference (MCID) for ATRS was achieved at 12 months onwards. Conclusions: Surgical management of CATR patients with FHL tendon transfer appears to improve functionality and subjective outcomes, supporting its use amongst the treatment modalities available.

## 1. Introduction

Despite being the strongest and thickest tendon of the human body, the Achilles (calcaneal) is the most frequently ruptured tendon [1]. Achilles tendon ruptures (ATRs) are reported to have an annual incidence of 3.2–8.0 per 100,000 people in the United States [2]. ATRs occur more frequently in males compared to females with an incidence rate ratio of 3.5 [2]. Misdiagnosis or incorrect treatment can lead to CATR. Given that ATRs are susceptible to delays in diagnosis, operative treatments ranging from end-to-end repair to flexor hallucis longus (FHL) tendon transfers are potential treatment options for chronic Achilles tendon rupture (CATR) [3].

Owing to the increased gap between tendon ends, different surgical techniques have been developed to address CATR. FHL transfer for CATR repair was first suggested in 1991 [4]. In 1993, a modified approach of FHL tendon transfer was introduced, utilizing calcaneal anchoring [5]. Compared with alternative operative approaches, FHL tendon transfer initially demonstrated no re-rupture of the Achilles tendon and minor losses to the passive range of flexion of the hallux interphalangeal joint and active flexion of the second and third toe [5]. Despite the FHL being a strong, long tendon that contracts with the gastrocnemius–soleus complex, its functional outcomes and efficacy in active individuals are still debated [6]. Greater toe flexion stability for ground grip has been a concern with the long FHL harvest technique; post-operative therapy targeted towards strengthening of flexor hallucis brevis (FHB) and flexor digitorum longus (FDL) led to few functional deficits [7]. In cases of CATR with atrophic and retracted tendon stumps, operative treatment provides a better functional outcome for the patient [5].

Despite numerous treatment modalities available, the optimal treatment strategy remains debatable, given the limited high-quality evidence [8]. For Achilles tendon (AT) defects between 3 and 6 cm, local tendon transfer including the peroneus brevis (PB) and FHL tendon can be considered. For defects exceeding 6 cm, ipsilateral semitendinosus (ST) tendon can be utilized [8]. No conclusive evidence exists on the FHL being better than the PB in tendon transfers for CATR treatment; additionally, further augmentation has not demonstrated further positive functional outcomes [8]. A slower return to sport was witnessed in PB tendon transfer patients compared to FHL transfer patients, but a higher percentage of PB transfer patients eventually returned to sport compared to FHL transfer patients [9]. Current literature reports an overall complication rate for FHL tendon transfers of 14.8%, with major complications such as deep infection, deep vein thrombosis (DVT) and re-rupture following FHL tendon transfer [10]. Conversely, identified benefits include a beneficial compensatory FHL hypertrophy following grafting [11]. Nevertheless, the presence of non-validated outcome measures limits comparisons of CATR treatment.

The current meta-analysis is an update on FHL tendon transfer alone for the treatment of CATR. The evaluation of functionality and overall complications of FHL transfer alone in the long term is essential for future consensus on CATR treatment. Hence, this meta-analysis aims to assess the outcomes of FHL transfer for CATR. Isolated FHL transfer for CATR demonstrates improved functional outcomes and low overall complication rates, contributing to existing evidence and support for future consensus on CATR management.

## 2. Materials and Methods

### 2.1. Search Strategy and Data Sources

This review adhered to the Preferred Reporting Items for Systematic Reviews and Meta-analyses (PRISMA) guidelines [12]. A search was performed across multiple databases from 1946 to 31 July 2025, without language restriction. The databases searched include Ovid MEDLINE^®^, EMBASE (Elsevier), Cochrane Central Register of Controlled Trials and Cochrane Database of Systematic Reviews. With input from the lead author, the search strategy was developed and executed by a medical reference librarian. Vocabulary supported by keywords was used to extract studies mentioning chronic Achilles rupture or tendon injuries with flexor hallucis longus or autograft. The actual search strategy used with the search terms can be found in Supplementary Item: Item S1. This review was prospectively registered with the International Prospective Register of Systematic Reviews (PROSPERO: CRD42023489724)

### 2.2. Eligibility Criteria and Quality Assessment

Eligible randomized prospective or retrospective observational studies had to meet the following inclusion criteria: (1) patients of any age; (2) chronic Achilles tendon rupture repair with only a flexor hallucis longus tendon transfer; (3) reporting on outcomes; (4) if multiple studies were reported by the same institution or authors, the analysis included either the higher quality study or the most recent publication. Excluded were studies with literature not reporting primary outcomes, unpublished data, data published only in abstract form, non-full-length articles, case reports, review articles, conference abstracts, letters to the editor, abstracts and articles not translatable to English. Non-English articles were machine-translated using Google Cloud Translation. Six independent assessors divided the pool of articles and conducted the screening alongside data extraction. Any disagreements were discussed amongst co-authors and resolved by the first author, YJK. Six independent assessors evaluated the quality of included studies using the ROBINS-I Risk of Bias assessment Scale [13]. For included studies with multiple arms, only the arm receiving an intervention of FHL alone for CATR was extracted, whilst other arms were disregarded.

### 2.3. Outcomes

The outcomes extracted were the American Orthopedic Foot and Ankle—Ankle Hindfoot Score (AOFAS-AH), the Achilles Tendon Rupture Score (ATRS), and complications. The AOFAS-AH measures treatment outcomes for complex ankle or hindfoot injuries on a 100-point scale, where higher scores represent better outcomes [14]. The ATRS evaluates symptom severity and functional limitations on a scale of 0 to 10 with 10 items. A total maximum score of 100 is possible; higher scores indicate better outcomes [15]. Complications (*n* of events) were extracted at the final follow-up.

### 2.4. Data Extraction/Outcome and Minimum Clinically Important Difference (MCID) Interpretation

AOFAS-AH were extracted in the following epochs: pre-operatively (baseline), 6 months and ≥12 months post-operatively. Similarly, ATRS were extracted pre-operatively (baseline) and ≥12 months post-operatively. Complications (*n* of events) were evaluated at final follow-up. A minimum clinically important difference (MCID) for ATRS was set at 10 points [15], utilizing ATRS values for acute Achilles tendon ruptures. Following ATRS, AOFAS-AH is the second most reported outcome in literature involving Achilles tendon ruptures [16]. No clear MCID exists for AOFAS-AH, and limited literature exists in translating AOFAS-AH to the functional status of the patient [17].

### 2.5. Statistical Analysis

Analysis was conducted as a single-arm meta-analysis, with no comparator group. The means of continuous variables and proportions in our study were pooled using the random-effect generic inverse variance method of DerSimonian and Laird [18]. The heterogeneity of effect size estimates across the studies was quantified using the Q statistic and I^2^. A value of I^2^ of 0–25% indicates minimal heterogeneity, 26–50% moderate heterogeneity and 51–100% substantial heterogeneity [19]. Moreover, a leave-one-out sensitivity analysis was conducted to assess individual study weight on pooled outcomes by excluding one study at a time. Publication bias was assessed using a funnel plot, where possible [20]. Authors were contacted three times via email to obtain any relevant information that was unavailable in published articles. If contact was unsuccessful, either the data set for that outcome or the study itself was removed from analysis due to the inability to pool. Data meta-analysis for means, standard deviation (SD) and proportions was performed using CEBM analyst software (Open Meta v0.24.1, Brown University, Providence, RI, USA).

## 3. Results

### 3.1. Study Selection and Characteristics

**Figure 1:**
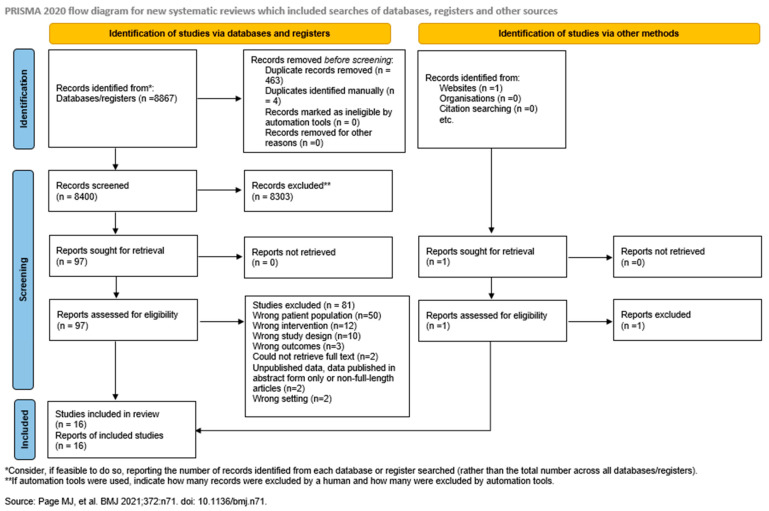
PRISMA flowchart detailing the selection process [21,22,23,24,25,26,27,28,29,30,31,32,33,34,35,36,37].

Sixteen studies were included from the initial search of 8867 potential articles. The PRISMA flowchart in Figure 1 details the selection process. Seven studies were retrospective case series [21,22,23,24,25,26,27,28], four were prospective case series [29,30,31,32], three were retrospective cohort studies [33,34,35] and one was cross-sectional [36]. A total of 323 patients met the inclusion criteria, with 68.7% (*n* = 222) being male and 31.3% (*n* = 101) being female. The mean age ranged from 39.5 to 70.0 years (95% CI: 49.96, 60.82, I^2^: 96%) [21,22,23,24,25,27,28,29,30,31,32,33,34,35,36]. Average defect length reported in patients was 5.93 cm (95% CI: 4.86, 7.01, I^2^: 97%) [21,22,23,25,28,29,31,32,35,36]. Elongated ATs were debrided, leaving the healthy AT fibers in nine studies [21,22,23,25,29,31,32,35,36]. Average time to surgery was 14 weeks (95% CI: 10.98, 17.04, I^2^: 95%) [21,22,23,25,29,31,32,35,36]. Our meta-analysis included 23 patients with reported co-morbidities, such as diabetes mellitus, arterial hypertension and unspecified thyroid disease [22,30,35]. Other reported co-morbidities included gout and chronic renal failure [33,35]. Baseline characteristics can be found in Table 1. Out of the sixteen studies, eight studies operated using a single incision technique [22,24,25,29,31,32,35,36], six studies were operated using a double incision technique [21,23,26,30,33,34] and one study used both single and double incision techniques [27]. Five of the studies harvested FHL at the plantar aspect of the foot [21,26,33,34,36]. Ten studies harvested FHL at the posterior aspect of the foot [22,23,24,25,28,29,30,31,32,35]. One study utilized FHL harvested at the posterior aspect of the ankle and the plantar aspect of the foot [27]. In our meta-analysis, post-operative rehabilitation included a moveable brace and casting for 4–8 weeks +/− weight-bearing. Initially, a non-weight-bearing cast was implemented in four studies [21,32,34]. Physiotherapy was part of post-operative rehabilitation in nine studies [21,22,26,29,31,33,35,36]. Further operative and post-operative rehabilitation characteristics can be found in Table 2.

### 3.2. Risk of Bias

**Figure 2:**
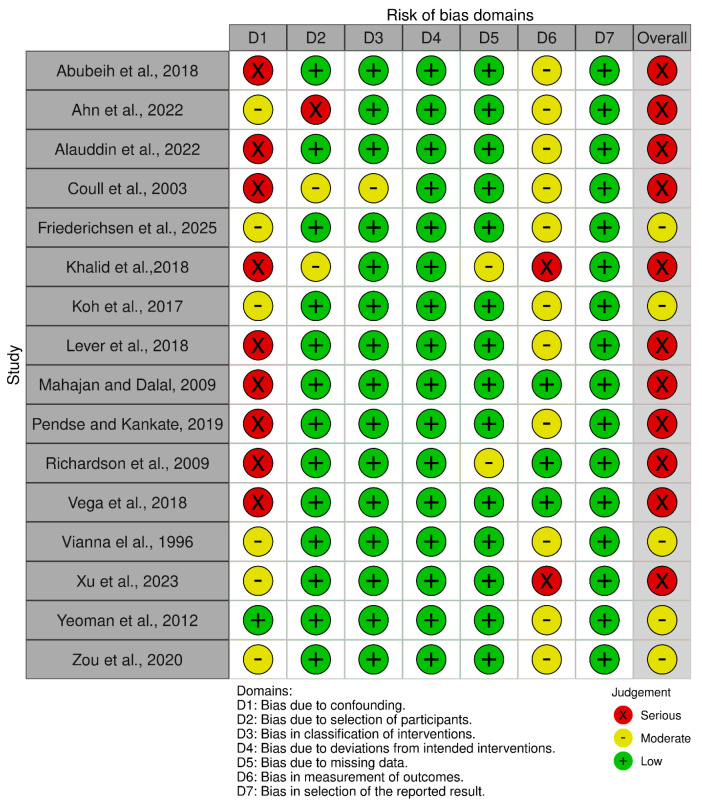
ROBINS-I risk of bias quality assessment scale [21,22,23,24,25,26,27,28,29,30,31,32,33,34,35,36].

The results of the quality assessment of the sixteen included studies can be found in Figure 2. All studies were observational and assessed using the ROBINS-I tool. Of the sixteen included studies, eleven had a serious risk of bias [22,23,24,25,26,27,31,32,33,34,36]. The remaining five were at moderate risk [21,28,29,30,35]. Serious risk of bias was found in the domains of confounding, selection of participants and measurement of outcomes.

### 3.3. AOFAS-AH

The AOFAS-AH was reported in ten studies, including 221 patients, in Table 3. Mean estimates, confidence intervals and heterogeneity (I^2^) values were all extracted from a forest plot. At baseline [21,23,25,29,31,32,33,35,36,38] the mean pooled score was 56.85 (95% CI: 51.03, 62.68, I^2^ = 96%). At 6 months post-operatively [29,31,35], the mean pooled score for 61 patients was 91.17 (95% CI: 89.37, 92.97, I^2^ = 2%). At ≥12 months post-operatively [21,22,23,25,26,32,33,34,35], the mean pooled score for 191 patients was 91.46 (95% CI: 88.45, 94.48, I^2^ = 93%). The mean AOFAS-AH score for open technique at ≥12 months post-operatively was 90.39 (95% CI: 86.46, 94.32, I^2^ = 95%) [22,25,26,32,33,35], whereas the mean AOFAS-AH score for endoscopic technique at ≥12 months post-operatively was 93.50 (95% CI: 90.80, 96.21, I^2^ = 37%) [21,23,34]. Baseline AOFAS-AH demonstrated relative asymmetry of the forest plot. The inclusion of fewer than 10 studies at each follow-up point post-operatively limited distinguishing chance from real asymmetry through visual inspection of the funnel plots. The AOFAS-AH score leave-one-out sensitivity analysis can be found in Supplementary Figure S3.

### 3.4. ATRS

The ATRS was reported in three studies, including 75 patients, in Table 4. Mean estimates, confidence intervals and heterogeneity (I^2^) values were all obtained from a forest plot. The mean ATRS pre-operatively [21,22,36] was 31.04 (95% CI: 5.80, 56.28, I^2^ = 99%). At ≥12 months post-operatively [21,22,34], the mean score for 67 patients was 90.73 (95% CI: 83.69, 97.77, I^2^ = 89%). The inclusion of fewer than 10 studies at each follow-up point post-operatively limited distinguishing chance from real asymmetry through visual inspection of the funnel plots. The ATRS score leave-one-out sensitivity analysis can be found in Supplementary Figure S4.

### 3.5. Complications

Complications were reported in thirteen studies in Table 5. The top three recorded complications include superficial infection, limitation of activity and disturbed wound healing. Superficial infection was reported at 4.2% (95% CI: 0.01, 0.07, I^2^ = 0%) [23,25,29,32,33,34,35,36]. The limitation of activity was reported at 4.0% (95% CI: 0.01, 0.08, I^2^ = 0%) [23,27,28,30,32,33]. The disturbed wound healing rate was reported at 3.8% (95% CI: 0.01, 0.06, I^2^ = 0%) [22,23,25,26,28,29,30,33,34,35]. Disturbed wound healing demonstrated relative symmetry in the forest plot. The complications’ leave-one-out sensitivity analysis can be found in Supplementary Figure S5. Funnel plots for all outcomes can be found in Supplementary Figure S6.

## 4. Discussion

Through the findings of this meta-analysis, FHL tendon transfers alone demonstrate sequentially improving functionality in CATR patients from operation to 12 months and beyond, as seen by AOFAS-AH and ATRS. Our meta-analysis evaluated isolated FHL tendon transfers without additional grafting techniques or V-Y lengthening. MCID is seen to be achieved for ATRS by the final follow-up. Furthermore, FHL tendon transfers demonstrate a low number of overall complications at 7.5%. The use of FHL tendon alone, therefore, continues to be a viable mainstay in the treatment repertoire available to surgeons. The findings of this current meta-analysis provide the groundwork for future randomized trials to directly compare different surgical modalities against FHL tendon transfer in a bid to find optimal treatment for patients suffering from CATR.

CATRs pose a clinical challenge in restoring the biomechanics of the ankle and foot, given that the medial gastrocnemius (MG) experiences an instant 15% reduction in fascicle length compared to the contralateral healthy limb [39]. Consequently, to the concerns of gastrocnemius plasty procedures, local autologous tendon transfers have been used in the reconstruction of CATRs [40]. Owing to the relatively long (10–12 cm) tendon length and its observed compensatory hypertrophy [11], existing literature indicates that the FHL offers a biomechanical advantage to the ankle and foot function. This is supported by a previous meta-analysis, which compared FHL tendon transfer with FHL augmentation, demonstrating an improvement in AOFAS-AH scores from 57.09 pre-operatively to 92.97 post-operatively in the FHL-only group [41]. The FHL-augmented group reported a superior AOFAS-AH score of 95.25 post-operatively [41]. This could be attributed to a higher pre-operative score in the FHL augmentation group. Similarly, an additional meta-analysis reported a pre-operative score of 54.7, which increased to 90.8 post-operatively. Our results are concordant with these previous findings, as we also witnessed an improvement in AOFAS-AH scores at 6 months and ultimately 12 months onwards. It is postulated that the improvement in functional outcomes is attributable to the FHL being a strong plantar flexor and that its axis of contractile force closely reproduces that of the Achilles tendon [42]. Moreover, augmentation techniques, such as V-Y plasty and fascial turndown flap, produce large wounds that impact the biomechanics of the gastrocnemius–Achilles tendon complex [8].

To further assess post-operative functionality, ATRS scores were also evaluated in our meta-analysis. A study additionally reported an improved post-operative ATRS score of 89.9 ± 7 [3]. We similarly found that ATRS scores improved, and MCID was achieved 12 months onwards. Common autologous tendon transfers utilized alongside FHL include hamstring and peroneus brevis (PB) autografts. The study further compared the ATRS of an FHL tendon transfer with ST autografts and PB transfer. Post-operative ATRSs achieved with both PB and ST autografts were 92.3 ± 2.2 and 91.3 ± 2.12, respectively [3]. The post-operative ATRSs in both PB and ST autografts were superior to the score achieved by the FHL within their study. However, these superior outcomes could be attributed to the higher pre-operative ATRS scores for the ST and PB groups compared to a pre-operative score of 38.2 ± 20.5 recorded in the FHL group [3]. In a study evaluating gap defects < 6 cm, the FHL achieved a post-operative ATRS of 88.9 ± 3.1, similar to the post-operative score of 89.5 ± 3.1 reported with PB transfers [43]. For most orthopedic surgeons, the FHL tendon remains as a workhorse in tendon transfer for CATR [40]. The improvement in ATRS functional scores was attributed to the increased vascularity of the FHL’s muscle belly and its ability to maintain normal ankle muscle balance [10]. Apart from the triceps surae, the FHL generates plantar flexion around the heel similar to the AT. In an injured limb, the relative contribution to force production capacity after ATR is highly attributable to the FHL in regard to the decrease in the MG [44]. In contrast to the FHL, the use of an evertor, such as the PB, results in less functional repair for the plantar flexion role of the AT [45].

In addition to functional outcomes, complications are a useful parameter in selecting the appropriate treatment modality for CATR. Analyzing commonly reported complications in existing literature and previous meta-analyses, our results indicated a lower overall complication rate than that previously reported [3,41,46]. It is not uncommon for individuals with CATR to present with etiologically significant co-morbidities. These include poor exercise conditioning, metabolic co-morbidities, advanced age or history of corticosteroid or fluoroquinolone use [47]. CATR patients with these co-morbidities often have problems with disturbed wound healing and tissue repair, which is further exacerbated by the avascular nature of the typical site of ATR, the midsection [47]. Due to triceps surae complex contracture and long muscle tendon complex length with scar tissue, plantar flexion power and ankle stability can be compromised. The FHL is hypothesized to increase vascularity at avascular sites in the midsection of the repaired AT and better promote wound healing. A systematic review reported surgical wound infections as the most common complication, at a rate of 7.8%, higher than our finding of 4.2% [3]. The difference in complications can be explained by the inclusion of additional augmentable techniques such as V-Y plasty and gastrocnemius turndown flaps. In addition, the overall mean time to surgery was appreciably longer at 26.4 ± 22.6 weeks [3]. It was argued that several variables affect both functional outcomes and complications, including chronicity, tendon gap size, gender and age [3]. We found evidence to support this idea, as studies with the most reports of both superficial infection and disturbed wound healing were in the elderly population and with a longer time to surgery [33,34]. Another meta-analysis reported a higher overall complication rate of 11.6% than our 7.5% [41]. Variance of complication rates within the analyzed literature was argued to be due to the difference in baseline characteristics and heterogeneity rather than the difference in techniques of repair by FHL tendon transfers only [41]. Thus, identifying a particular baseline characteristic contributing to heterogeneity in complication rates is complex and requires further investigation. In an alternative review, free tendon grafts were utilized for the surgical treatment of main body AT tears. Like our meta-analysis, the most common complication was superficial post-operative infection, followed by wound dehiscence, with a prevalence of 3.5% and 2.2%, respectively [9]. Superficial infections in the study were observed in Achilles tendon allografts and semitendinosus and gracilis autografts [9]. Wound dehiscence was limited to AT allografts, xenografts and semitendinosus autografts augmented with additional FHL; in addition, sural nerve numbness and injury to the tibial nerve were reported [9]. In contrast, our meta-analysis with FHL transfer did not report any nerve injury or associated numbness. Moreover, nicotine dependence and smoking are associated with post-operative complications following ATR repair. In a study comparing non-nicotine and nicotine-dependent patients, the nicotine-dependent group reported a significantly higher risk for wound disruption and infection after ATR repair [48]. Nicotine interferes with oxygen supply through vasoconstrictive effects and the release of epinephrine, resulting in decreased perfusion and delayed wound healing [49]. Similarly, carbon monoxide and hydrogen cyanide in smoke induce hypoxia and impair wound healing [49]. In our meta-analysis, only four studies [22,28,30,34] reported on the smoking status of patients, with no clear association of smoking status with reported disturbed wound healing. Further exploration with regard to wound healing in CATR is recommended in the future.

Incidence of surgical site infection following ATR repair was more commonly observed without prophylactic antibiotic use and open incision technique, carrying implications for clinical practice [50]. In our meta-analysis, open incision techniques were more commonly used in our included studies [22,24,25,26,29,30,31,32,33,35,36]. The use of endoscopic FHL transfer was reported in three studies only [21,26,34]. A total of 7 out of 10 superficial infections were reported with open incision techniques [25,29,32,33,36]. In an alternative study, the majority of superficial post-operative infections were reported in open and mini-open techniques in free tendon grafts [9].

There are two incision techniques described for FHL transfer: single and double incision techniques. Single posteromedial incisions provide several advantages, including a faster and less challenging dissection of the midfoot and neurovascular structures near the plantar surface, not being a concern [51]. However, a notable drawback of the single incision is a shorter FHL harvest [52]. In the double incision technique, an additional incision is made in the midfoot. Current literature reporting on incision techniques and nerve injury as a complication is limited to cadaveric specimens. A study, evaluating single versus double incision techniques with FHL transfer in frozen dead specimens, yielded 5.16 cm and 8.09 cm long tendons, respectively. In our meta-analysis, the mean gap defect was 5.93 cm. Accounting for the atrophic distal stump in CATR, additional graft length is advantageous for the suturing and fixation of the FHL tendon transfer in calcaneal bone. With a double incision, the FHL tendon is harvested distally to the knot of Henry near the medial and lateral plantar nerve [53]. An alternative study raised that the retraction puts both the medial plantar nerve and lateral plantar nerve at risk for nerve injury [54]. The study substantiated this concern by reporting a total of eight injuries in 24 feet to both branches of the posterior tibial nerve—medial plantar nerve and lateral plantar nerve [55]. Moreover, it is argued that regardless of incision technique, medial and lateral plantar nerves did not occur more frequently, due to the large distance between the FHL and the medial and lateral plantar nerves [56]. In our analysis, eight included studies utilized a double incision technique, and none of those studies reporting nerve injury recorded any cases [21,23,26,27,28,30,33,34]. As such, we cannot draw an association between incision technique and potential nerve injury. Further exploration of surgical incision techniques regarding neurovascular bundle injury is recommended for future studies.

To our knowledge, there is no clear post-operative rehabilitation protocol for CATR. Early range of motion of the knee is advised to avoid the limitations of joint immobility, and patients are encouraged to bear as much weight as possible on the second day after surgery, aided by a below-the-knee cast and elbow crutches [8]. A study evaluated early mobilization and resumption of activity after ATR V-Y surgical repair. The author concluded that surgical repair, regardless of acute or chronic rupture, combined with early mobilization, reduces the range of motion loss and allows for resumption of normal activity, including stair climbing or regular exercise, within 3.3 months. Moreover, it was suggested that repetitive tensile loading after surgical loading prevents adhesion formation and improves vascularity, minimizing activity limitation and loss of ankle range of motion [57]. However, this study utilized an augmented technique with V-Y plasty rather than an FHL transfer. In a longitudinal study comparing early mobilization and cast immobilization, the early mobilization group had a faster return to physical activity at 5.1 months post-operatively, compared to 6 months in cast-immobilized patients [58]. It was hypothesized that early weight-bearing with cyclic tension on both the gastrocsoleus muscle complex and the site of tendon repair gives an appropriate stimulus for growth and repair [58]. However, the difference in early mobilization reported was regarded as insignificant and reported solely on acute ATR. A clinical consensus statement on acute Achilles tendon pathology stated that care routinely consists of progressing the patient to weight-bearing and rehabilitation an average of 14 to 21 days post-operatively, or ideally, after the sutures heal [59]. In our meta-analysis, six studies reported on activity limitation, stating a rate of 4% in 119 patients following CATR repair [23,27,28,30,32,33]. Mobilization rehabilitation included a moveable ankle brace at 4 to 8 weeks post-operatively, with weight-bearing concurrently. It was difficult to establish if post-operative rehabilitation meaningfully impacts the incidence or severity of post-operative activity limitation.

### Limitations

As with all meta-analyses, limitations are present. The primary limitation is that all included studies are observational designs. The lack of randomized controlled trial (RCT) studies inevitably leads to selection bias and confounders. Significant confounders include BMI, age, pre-injury activity levels, history of blunt trauma to the Achilles tendon or autoimmune arthritis, and fluoroquinolone and steroid use. The majority of included studies reported on age [21,22,24,25,26,27,29,30,33,34,35,36]. Steroid usage was limited to three studies [22,28,30]. BMI was reported in six studies [23,27,28,30,32,33]. Confounders were reported; however, not controlled for. When calculating the overall complication rate, each patient was assumed to account for one event, provided the number of individual complications did not exceed the overall study sample size. A leave-one-out sensitivity analysis was conducted in outcomes with more than three studies analyzed. The sample size in our meta-analysis was rather small (*n* = 323), limiting the power of this analysis. Moreover, post-operative rehabilitation protocols and time of mobilization were not analyzed as part of the outcome evaluation. Existing literature reports solely on acute rupture patients with early functional mobilization over one year. Consequently, a standardized protocol or guideline for post-operative care or treatment remains a question. Literature regarding post-operative treatment is limited to acute Achilles tendon ruptures over short-term follow-up; thus, eliminating the ability to evaluate and appraise post-operative mobilization protocols within our analysis. Literature reporting on patient baseline confounders in CATR is limited. To the best of our knowledge, existing research only reports on general risk factors for ATR or baseline confounders related to acute ATR. It could be that metabolic co-morbidities, including hypercholesterolemia, obesity, diabetes mellitus and thyroid disease, significantly predicted the risk of post-operative complications in acute Achilles tendon ruptures. Hence, identifying risk factors for CATR is important. Reporting was limited to acute rupture patients or small sample size studies, limiting any generalization of these findings to hindering our generalization of findings regarding co-morbidities and post-operative complications in CATR patients. Furthermore, existing heterogeneity in our sample size, with ages ranging from 39.5 to 70 years old, limited the feasibility of drawing any association between age and re-rupture, similar to previous literature. Heterogeneity was additionally present in the mean surgery time of the sample size. Additionally, our meta-analysis relied on both patient- and physician-reported outcome measures, including ATRS and AOFAS-AH scores. Objective outcome measurements, including electromyography and pedographs of the injured limb versus the uninjured limb, were not reported on. ATRS remains as the only specific outcome measure for ATRs [15]; however, a signification portion of the score relates to resumption of activities, which limits applicability to older populations. Finally, minimum clinically important differences utilized in this meta-analysis are based on data for acute Achilles tendon ruptures.

## 5. Conclusions

The current study is an updated meta-analysis exploring the use of FHL tendon transfer only without any supplementary grafting as an intervention for CATR. The results of FHL tendon transfer demonstrate improvement in functionality with respect to AOFAS and ATRS, and a low overall complication rate. FHL tendon transfer only demonstrated no cases of nerve injuries or re-ruptures. Despite the increase in patient functionality scoring, the sample size analyzed remains small. Additionally, literature gaps in regard to CATRs are abundant and require further exploration to develop a more standardized approach to optimal treatment modalities, evaluate outcomes and develop a standardized treatment protocol. Further studies, such as randomized controlled trials (RCTs), including a greater sample size and medical consensus would further emphasize the results of this study.

## Figures and Tables

**Table 1:** Baseline characteristics of included studies.

Study	Study Design	Sample Size (*n*)	Mean Age ± SD (Years)	Mean Defect Length ± SD (cm)	Mean Injury Time ± SD (Weeks)
Abubeih, 2018 (Egypt) [32]	Prospective case series	21	40 ± 18	7 ± 2	9 ± 4
Ahn, 2022 (Korea) [36]	Cross-sectional	28	51 ± 14	3 ± 1	13 ± 20
Alauddin, 2022 (Bangladesh) [31]	Prospective case series	21	39 ± 14	7 ± 1	11 ± 7
Coull, 2003 (Ireland) [27]	Retrospective case series	12	61 ± 9	NR	NR
Friederichsen, 2025 (Switzerland) [28]	Retrospective case series	28	61 ± 12	10 ± 3	NR
Khalid, 2019 (USA) [26]	Retrospective case series	10	58 ± NR	NR	NR
Koh, 2017 (Singapore) [35]	Retrospective cohort	29	56 ± 13	5 ± 2	17 ± 13
Lever, 2018 (UK) [34]	Retrospective cohort	20	60 ± 12	NR	28 ± 35
Mahajan and Dalal, 2009 (UK) [33]	Retrospective cohort	36	70 ± 5	NR	15 ± 3
Pendse and Kankate, 2019 (UK) [25]	Retrospective series	16	66 ± 10	4 ± 1	32 ± 17
Richardson, 2009 (USA) [24]	Retrospective case series	22	56 ± 9	NR	NR
Vega, 2018 (Spain) [23]	Retrospective case series	12	69 ± 6	6 ± 2	NR
Vianna, 1996 (Brazil) [30]	Prospective case series	10	54 ± 13	NR	8 ± 5
Xu, 2023 (China) [22]	Retrospective case series	28	46 ± 11	7 ± 2	15 ± 7
Yeoman, 2012 (UK) [29]	Prospective case series	11	53 ± 10	6 ± 2	27 ± 25
Zou, 2020 (China) [21]	Retrospective case series	19	47 ± 11.	6 ± 1	15 ± 4

SD: standard deviation; NR: not reported.

**Table 2:** Incision technique and post-operative rehabilitation with respect to mean follow-up time.

Study	Incision Technique	Post-Operative Rehabilitation	Mean Follow-Up Time (Years) ± SD
Abubeih, 2018 [32]	Single	Non-weight-bearing below-knee plaster	1 ± 0
Ahn, 2022 [36]	Single	Short leg cast, toe touch weightbearing and physiotherapy	5 ± 3
Alauddin, 2022 [31]	Single	Long leg anterior cast reduced to short leg anterior slab + physiotherapy	NR
Coull, 2003 [27]	Single and double	NR	5 ± 2
Friederichsen, 2025 [28]	Double	Immobilized in boot orthosis and physiotherapy	2 ± 0
Khalid, 2019 [26]	Double	Weight-bearing cast and physiotherapy	3 ± 1
Koh, 2017 [35]	Single	Backslab and physiotherapy	1 ± 0
Lever, 2018 [34]	Endoscopic double	Non-weight-bearing slab followed by a weight-bearing cast	6 ± 2
Mahajan and Dalal, 2009 [33]	Double	Below the knee cast and physiotherapy	1 ± 0
Pendse and Kankate, 2019 [25]	Single	Below the knee backslab with chemical prophylaxis	2 ± 1
Richardson, 2009 [24]	Single	NR	2 ± 2
Vega, 2018 [23]	Endoscopic double	Below the knee splint followed by weight bearing walker boot	3 ± 1
Vianna, 1996 [30]	Double	Padded compressive dressing and plaster cast followed by a cast boot	4 ± 1
Xu, 2023 [22]	Single	Below the knee plaster and physiotherapy followed by weight bearing brace	5 ± 2
Yeoman, 2012 [29]	Single	Below the knee plaster followed by weight bearing cast and physiotherapy	NR
Zou, 2020 [21]	Endoscopic double	Non-weight bearing cast followed by a physiotherapy	3 ± 0

SD: standard deviation, NR: not reported.

**Table 3:** American Orthopaedic Foot & Ankle Society—Ankle Hindfoot Score (AOFAS-AH).

AOFAS-AH Score	Mean Estimate	95% CI	I^2^	Included Study Groups (*n*)	Sample Size (*n*)
Pre-operative	56.85	51.03, 62.68	96%	10	221
6 months post-operative	91.17	89.37, 92.97	2%	3	61
≥12 months post-operative	91.46	88.45, 94.48	93%	9	191

CI: confidence interval.

**Table 4:** Achilles Tendon Rupture Score (ATRS).

ATRS Score	Mean Estimate	95% CI	I^2^	Included Study Groups (*n*)	Sample Size (*n*)
Pre-operative	31.04	5.80, 56.28	99%	3	75
≥12 months post-operative	90.73	83.69, 97.77	89%	3	67

CI: confidence interval.

**Table 5:** Reported complications.

Reported Complication	Proportion	95% CI	I^2^	Events/Sample Size (*n*)	Included Study Groups (*n*)
Overall complications	0.08	0.04, 0.11	40%	28/261	13
Superficial infections	0.04	0.0.1, 0.07	0%	10/173	8
Deep infections	0.02	−0.01, 0.05	0%	0/69	3
Disturbed wound healing	0.04	0.01, 0.06	0%	12/200	10
Re-rupture	0.02	−0.00, 0.04	0%	0/142	6
Activity limitation	0.04	0.01, 0.08	0%	4/119	6
Nerve injury	0.02	−0.01, 0.04	0%	0/108	5

CI: confidence interval.

## Data Availability

The data presented in this study are available on request from the corresponding author due to privacy.

## Supplementary Materials

The following supporting information can be downloaded at https://www.mdpi.com/article/10.3390/healthcare13212751/s1, Supplemental Item S1: Actual search strategies, Figure S1: AOFAS—AH score, Figure S2: ATRS score. Figure S3: AOFAS—AH leave-one-out analysis, Figure S4: ATRS leave-one-out analysis, Figure S5: Complications leave-one-out analysis, Figure S6: Funnel plots.

## Abbreviations

The following abbreviations are used in this manuscript:

CATR	Chronic Achilles tendon rupture
FHL	Flexor hallucis longus
FHB	Flexor hallucis brevis
FDL	Flexor digitorum longus
AOFAS-AH	American Orthopaedic Foot & Ankle Society—Ankle Hindfoot score
ATRS	Achilles tendon rupture score
MCID	Minimum clinically important difference
AT	Achilles Tendon
ATR	Achilles tendon rupture
MG	Medial gastrocnemius
ST	Semitendinosus
PB	Peroneus brevis
