# Supplementary material for: Isolated Flexor Hallucis Longus Tendon Transfer for Chronic Achilles Tendon Rupture: Systematic Review and Meta-Analysis
Source: Healthcare (Basel). 2025 Oct 30;13(21):2751. doi: 10.3390/healthcare13212751 (PMC12607451; doi:10.3390/healthcare13212751)
Supplement: Supplementary file 1 [file healthcare-13-02751-s001.zip › Supplementary Figure S1 AOFAS.pdf]

A

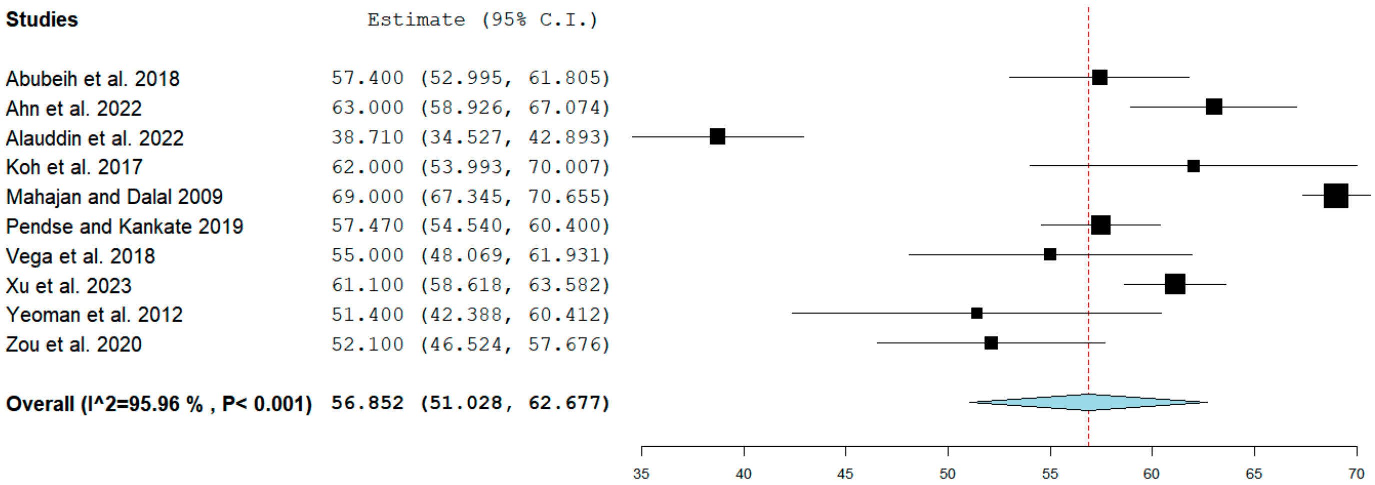
Studies
Estimate (95% C.I.)
Abubeih et al. 2018 57.400 (52.995, 61.805)
Ahn et al. 2022 63.000 (58.926, 67.074)
Alauddin et al. 2022 38.710 (34.527, 42.893)
Koh et al. 2017 62.000 (53.993, 70.007)
Mahajan and Dalal 2009 69.000 (67.345, 70.655)
Pendse and Kankate 2019 57.470 (54.540, 60.400)
Vega et al. 2018 55.000 (48.069, 61.931)
Xu et al. 2023 61.100 (58.618, 63.582)
Yeoman et al. 2012 51.400 (42.388, 60.412)
Zou et al. 2020 52.100 (46.524, 57.676)
Overall (I^2=95.96 % , P< 0.001) 56.852 (51.028, 62.677)
35
40
45
50
55
60
65
70

B

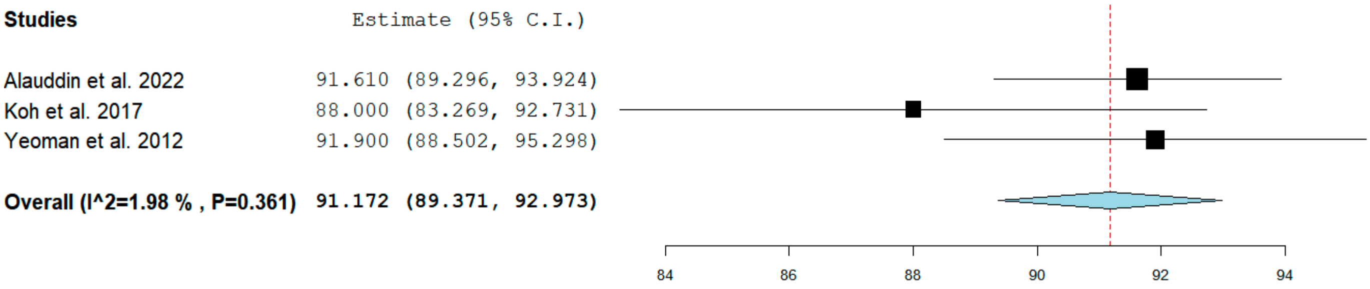
Studies
Estimate (95% C.I.)
Alauddin et al. 2022 91.610 (89.296, 93.924)
Koh et al. 2017 88.000 (83.269, 92.731)
Yeoman et al. 2012 91.900 (88.502, 95.298)
Overall (I^2=1.98 % , P=0.361) 91.172 (89.371, 92.973)
84
86
88
90
92
94

C

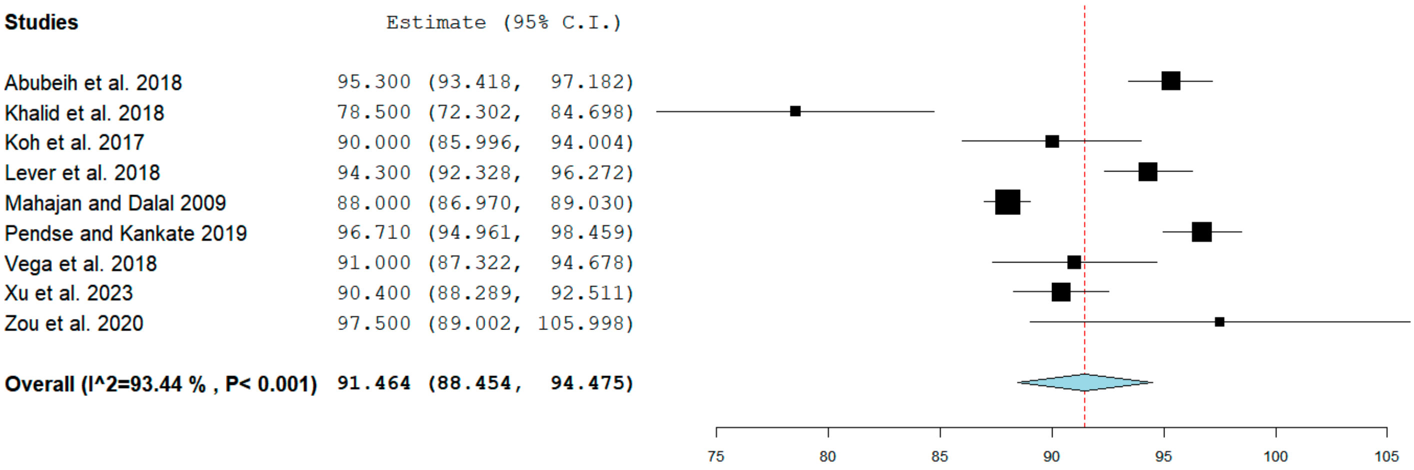
Studies
Estimate (95% C.I.)
Abubeih et al. 2018 95.300 (93.418, 97.182)
Khalid et al. 2018 78.500 (72.302, 84.698)
Koh et al. 2017 90.000 (85.996, 94.004)
Lever et al. 2018 94.300 (92.328, 96.272)
Mahajan and Dalal 2009 88.000 (86.970, 89.030)
Pendse and Kankate 2019 96.710 (94.961, 98.459)
Vega et al. 2018 91.000 (87.322, 94.678)
Xu et al. 2023 90.400 (88.289, 92.511)
Zou et al. 2020 97.500 (89.002, 105.998)
Overall (I^2=93.44 % , P< 0.001) 91.464 (88.454, 94.475)
75
80
85
90
95
100
105

D

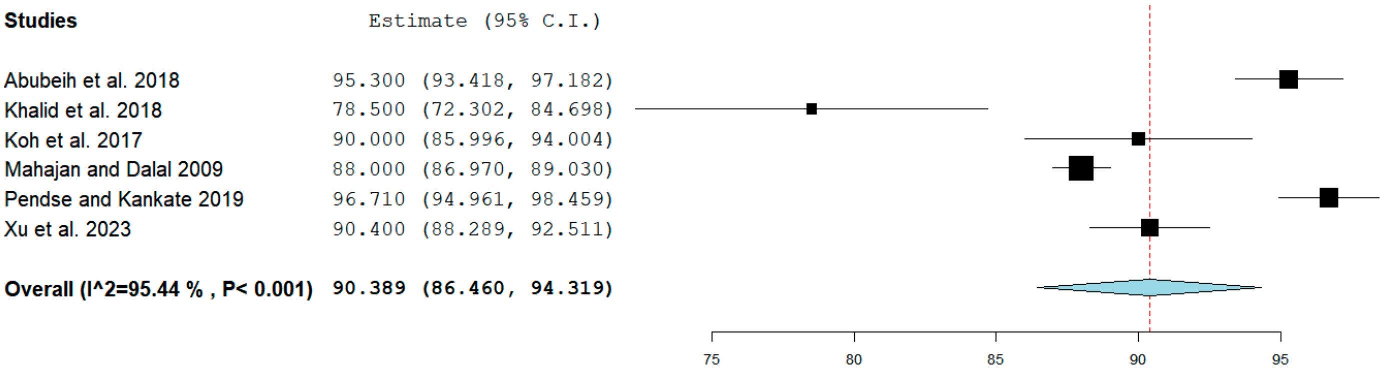
Studies
Estimate (95% C.I.)
Abubeih et al. 2018 95.300 (93.418, 97.182)
Khalid et al. 2018 78.500 (72.302, 84.698)
Koh et al. 2017 90.000 (85.996, 94.004)
Mahajan and Dalal 2009 88.000 (86.970, 89.030)
Pendse and Kankate 2019 96.710 (94.961, 98.459)
Xu et al. 2023 90.400 (88.289, 92.511)
Overall (I^2=95.44 % , P< 0.001) 90.389 (86.460, 94.319)
75
80
85
90
95

E

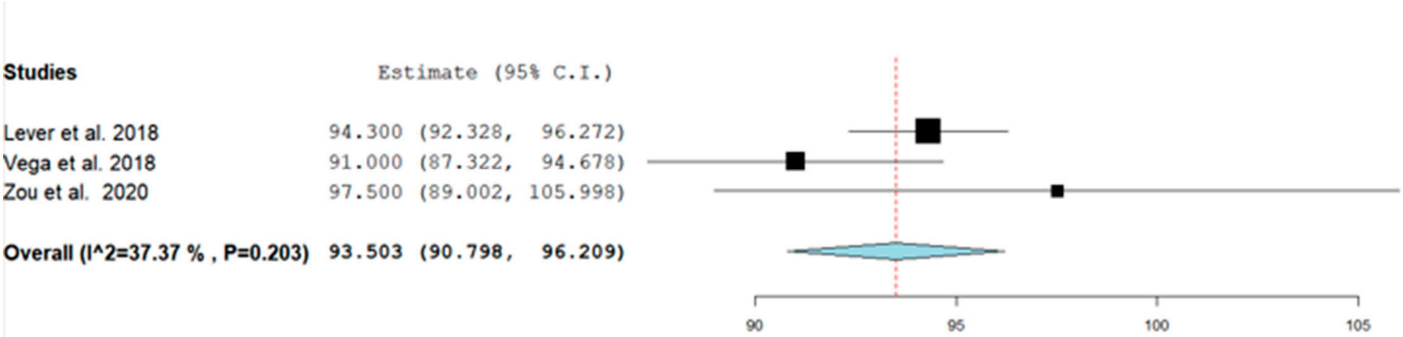

Supplementary Figure S1: Pooled estimate of American Orthopadic Foot & Ankle Society- Ankle Hindfoot score (AOFAS-AH) scores a) baseline, b) 6 months, c) ≥ 12 months, d)Open technique, e) endoscopic technique.
