# Supplementary material for: Isolated Flexor Hallucis Longus Tendon Transfer for Chronic Achilles Tendon Rupture: Systematic Review and Meta-Analysis
Source: Healthcare (Basel). 2025 Oct 30;13(21):2751. doi: 10.3390/healthcare13212751 (PMC12607451; doi:10.3390/healthcare13212751)
Supplement: Supplementary file 1 [file healthcare-13-02751-s001.zip › Supplementary Figure S2 ATRS.pdf]

A

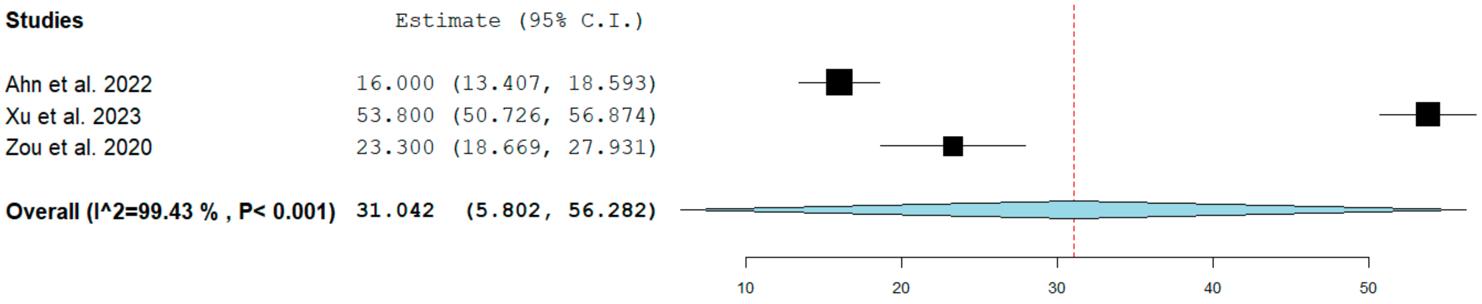

B

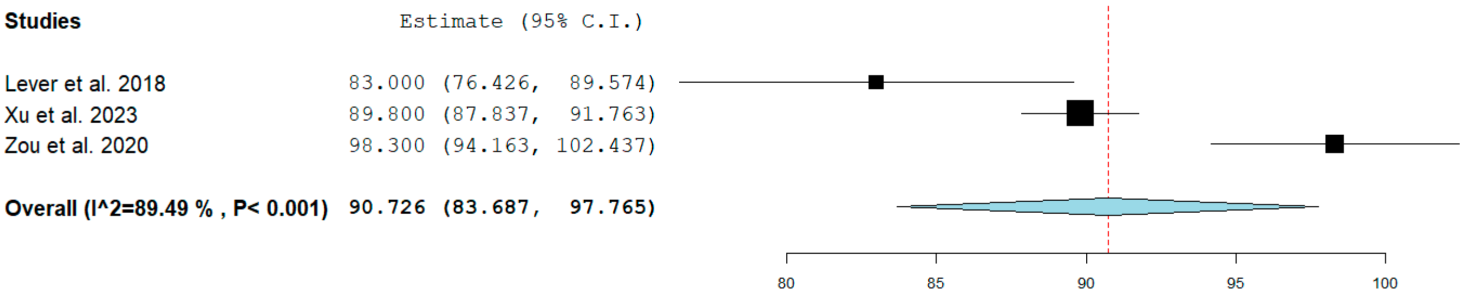

Supplementary Figure S2: pooled estimate of Achilles tendon rupture score (ATRS) scores a) baseline b) ≥ 12 months.
