# Supplementary material for: Isolated Flexor Hallucis Longus Tendon Transfer for Chronic Achilles Tendon Rupture: Systematic Review and Meta-Analysis
Source: Healthcare (Basel). 2025 Oct 30;13(21):2751. doi: 10.3390/healthcare13212751 (PMC12607451; doi:10.3390/healthcare13212751)
Supplement: Supplementary file 1 [file healthcare-13-02751-s001.zip › Supplementary Figure S3 AOFAS LEAVE ONE OUT.pdf]

A

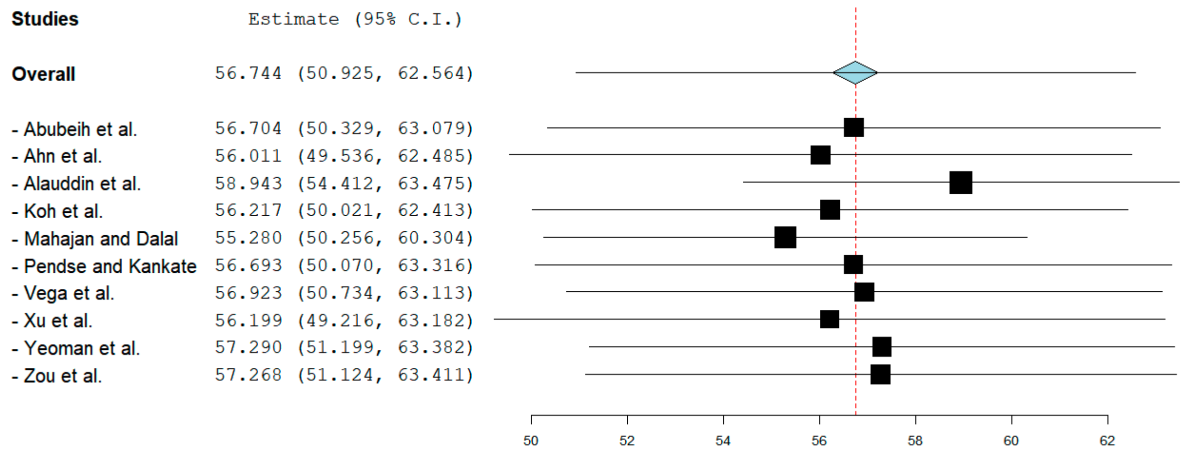

Studies
Estimate (95% C.I.)
Overall
56.744 (50.925, 62.564)
- Abubeih et al.
56.704 (50.329, 63.079)
- Ahn et al.
56.011 (49.536, 62.485)
- Alauddin et al.
58.943 (54.412, 63.475)
- Koh et al.
56.217 (50.021, 62.413)
- Mahajan and Dalal
55.280 (50.256, 60.304)
- Pendse and Kankate
56.693 (50.070, 63.316)
- Vega et al.
56.923 (50.734, 63.113)
- Xu et al.
56.199 (49.216, 63.182)
- Yeoman et al.
57.290 (51.199, 63.382)
- Zou et al.
57.268 (51.124, 63.411)
50
52
54
56
58
60
62

B

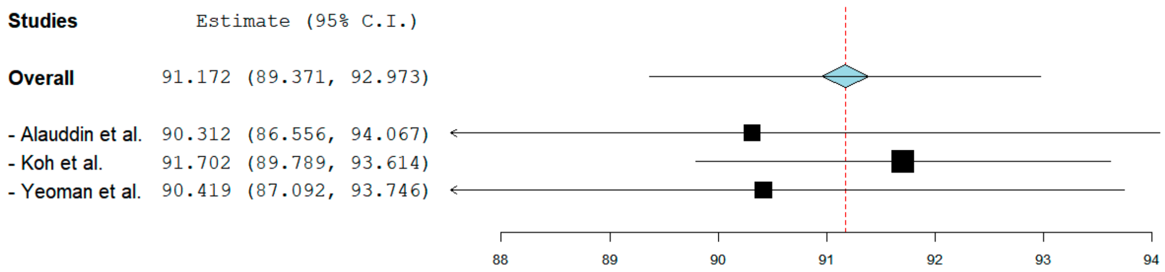

Studies
Estimate (95% C.I.)
Overall
91.172 (89.371, 92.973)
- Alauddin et al.
90.312 (86.556, 94.067)
- Koh et al.
91.702 (89.789, 93.614)
- Yeoman et al.
90.419 (87.092, 93.746)
88
89
90
91
92
93
94

C

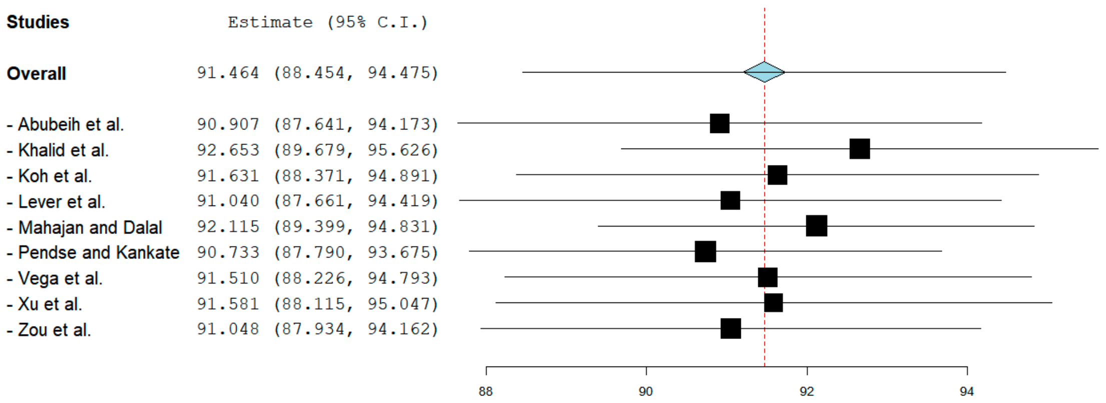

Supplementary Figure S3: American Orthopedic Foot and Ankle Society- Ankle Hindfoot Score (AOFAS-AH) leave-one-out analyses: A) Baseline, B)at 6 months and C)≥12 months.
