# Supplementary material for: Isolated Flexor Hallucis Longus Tendon Transfer for Chronic Achilles Tendon Rupture: Systematic Review and Meta-Analysis
Source: Healthcare (Basel). 2025 Oct 30;13(21):2751. doi: 10.3390/healthcare13212751 (PMC12607451; doi:10.3390/healthcare13212751)
Supplement: Supplementary file 1 [file healthcare-13-02751-s001.zip › Supplementary Figure S5 COMPLICATIONS LEAVE ONE OUT.pdf]

A

Studies	Estimate (95% C.I.)
**Overall**	0.075 (0.036, 0.114)
- Abubeih et al.	0.081 (0.037, 0.125)
- Ahn et al.	0.085 (0.040, 0.130)
- Coull et al.	0.081 (0.038, 0.124)
- Friederichsen et al.	0.073 (0.032, 0.115)
- Khalid et al.	0.072 (0.032, 0.111)
- Koh et al.	0.086 (0.044, 0.129)
- Lever et al.	0.060 (0.027, 0.093)
- Mahajan and Dalal	0.061 (0.025, 0.097)
- Pendse and Kankate	0.078 (0.036, 0.121)
- Vega et al.	0.076 (0.035, 0.118)
- Vianna	0.068 (0.031, 0.106)
- Xu et al.	0.085 (0.040, 0.130)
- Yeoman et al.	0.072 (0.032, 0.112)

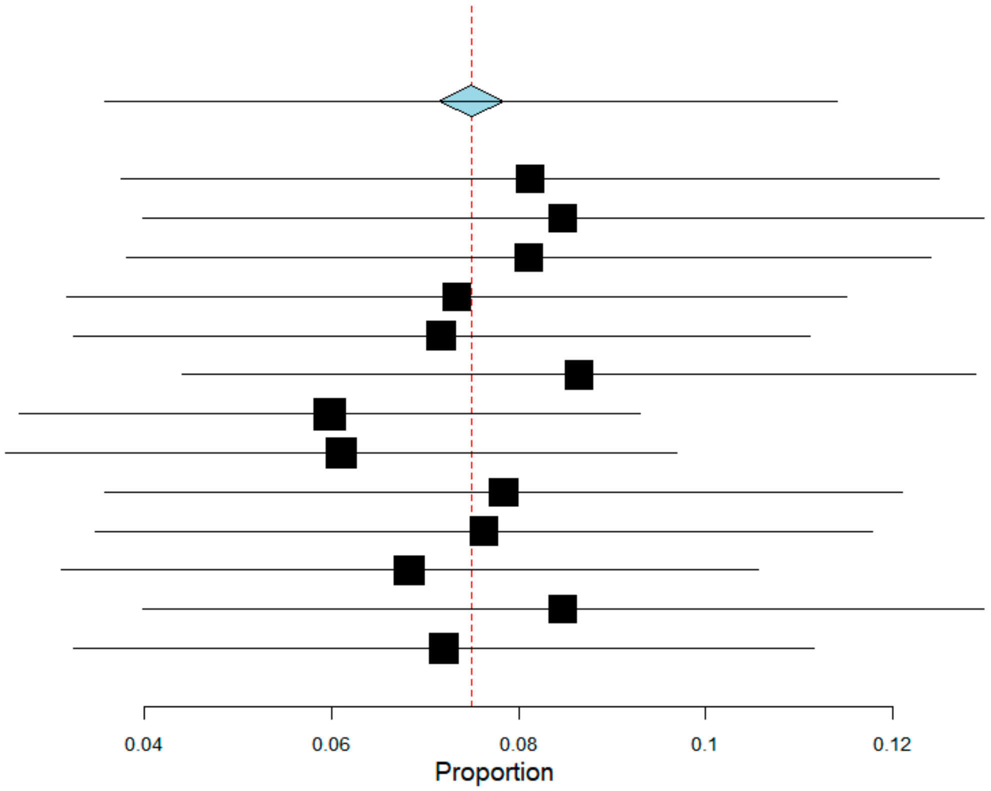

B

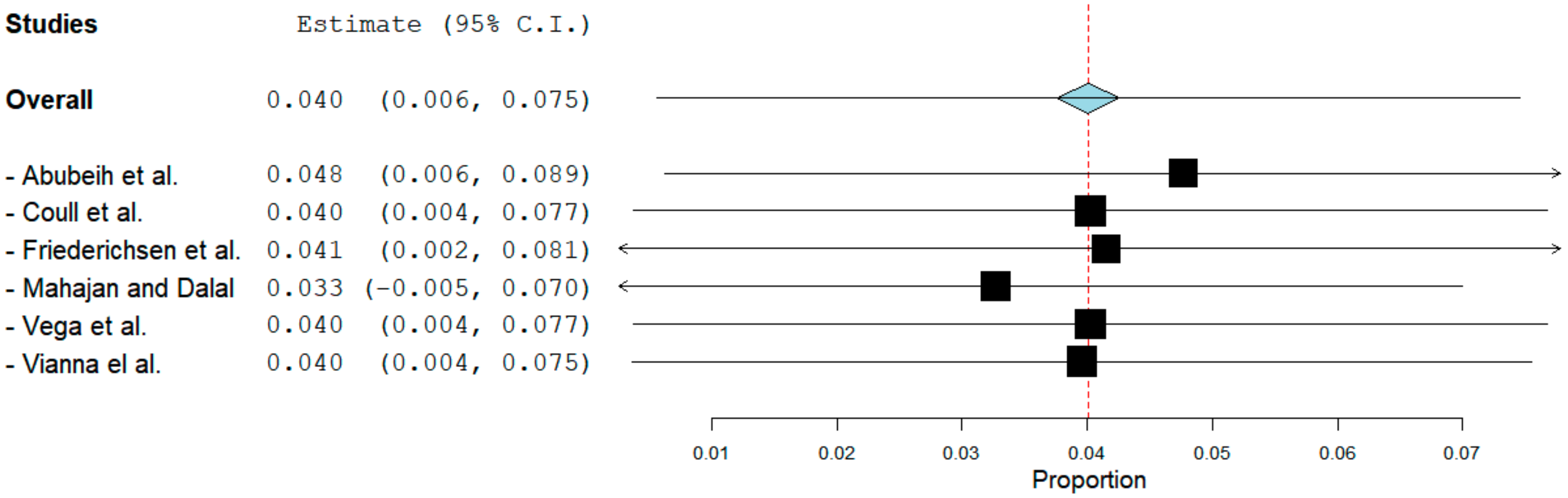
Studies
Estimate (95% C.I.)
Overall 0.040 (0.006, 0.075)
- Abubeih et al. 0.048 (0.006, 0.089)
- Coull et al. 0.040 (0.004, 0.077)
- Friederichsen et al. 0.041 (0.002, 0.081)
- Mahajan and Dalal 0.033 (-0.005, 0.070)
- Vega et al. 0.040 (0.004, 0.077)
- Vianna el al. 0.040 (0.004, 0.075)
0.01
0.02
0.03
0.04
0.05
0.06
0.07
Proportion

C

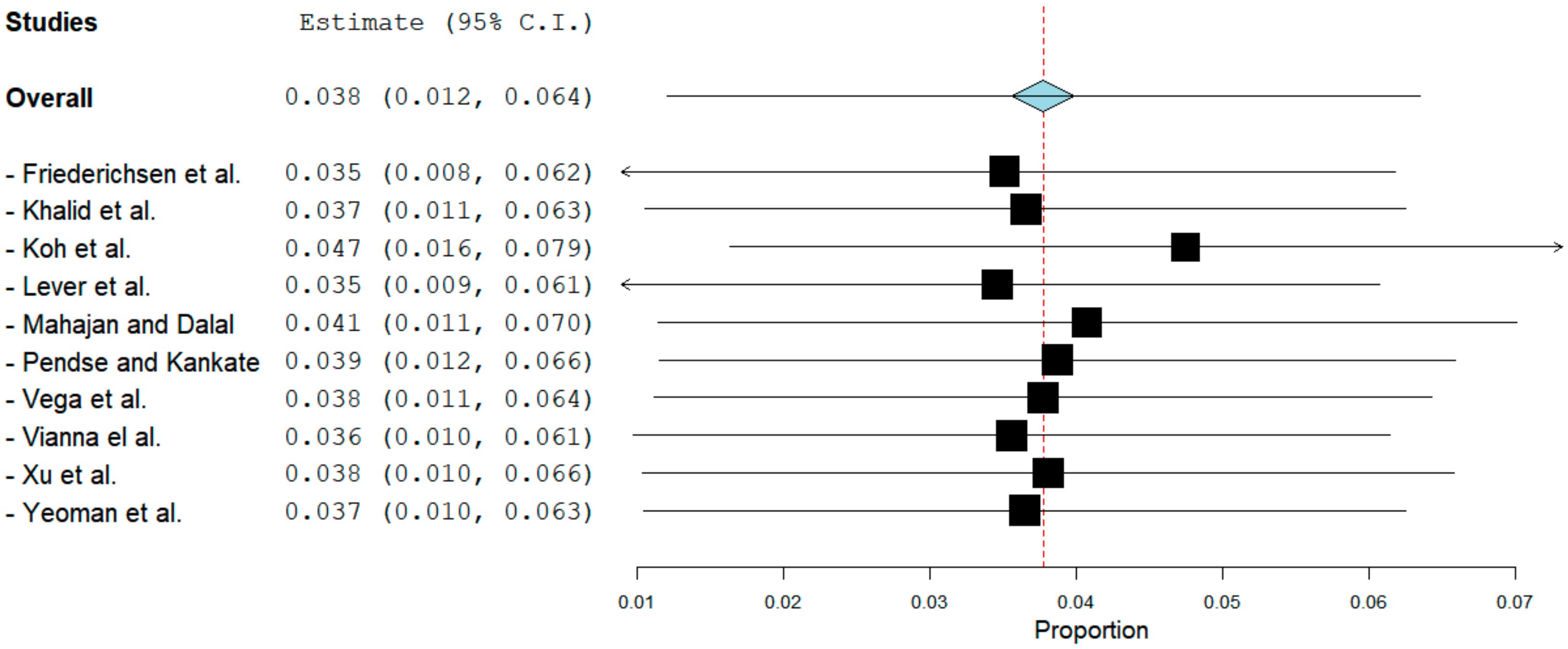
Studies
Estimate (95% C.I.)
Overall
0.038 (0.012, 0.064)
- Friederichsen et al.
0.035 (0.008, 0.062)
- Khalid et al.
0.037 (0.011, 0.063)
- Koh et al.
0.047 (0.016, 0.079)
- Lever et al.
0.035 (0.009, 0.061)
- Mahajan and Dalal
0.041 (0.011, 0.070)
- Pendse and Kankate
0.039 (0.012, 0.066)
- Vega et al.
0.038 (0.011, 0.064)
- Vianna el al.
0.036 (0.010, 0.061)
- Xu et al.
0.038 (0.010, 0.066)
- Yeoman et al.
0.037 (0.010, 0.063)
0.01
0.02
0.03
0.04
0.05
0.06
0.07
Proportion

D

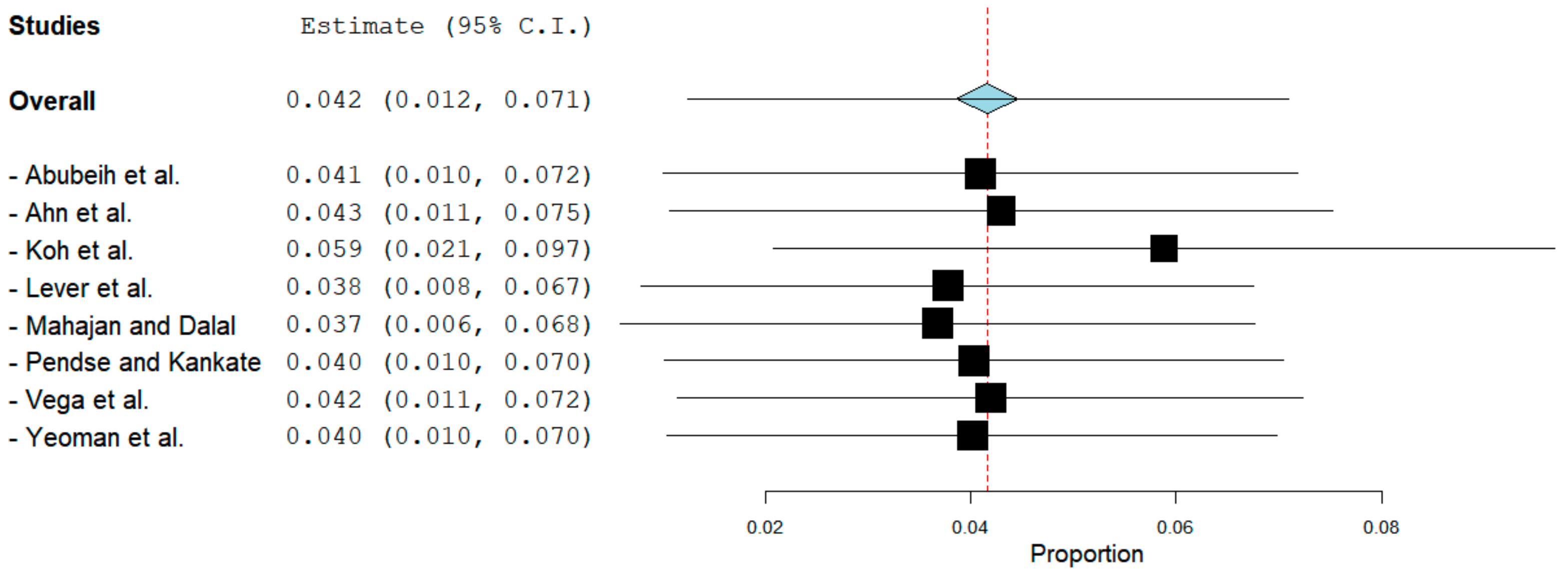
Studies
Estimate (95% C.I.)
Overall
0.042 (0.012, 0.071)
- Abubeih et al.
0.041 (0.010, 0.072)
- Ahn et al.
0.043 (0.011, 0.075)
- Koh et al.
0.059 (0.021, 0.097)
- Lever et al.
0.038 (0.008, 0.067)
- Mahajan and Dalal
0.037 (0.006, 0.068)
- Pendse and Kankate
0.040 (0.010, 0.070)
- Vega et al.
0.042 (0.011, 0.072)
- Yeoman et al.
0.040 (0.010, 0.070)
0.02
0.04
0.06
0.08
Proportion

E

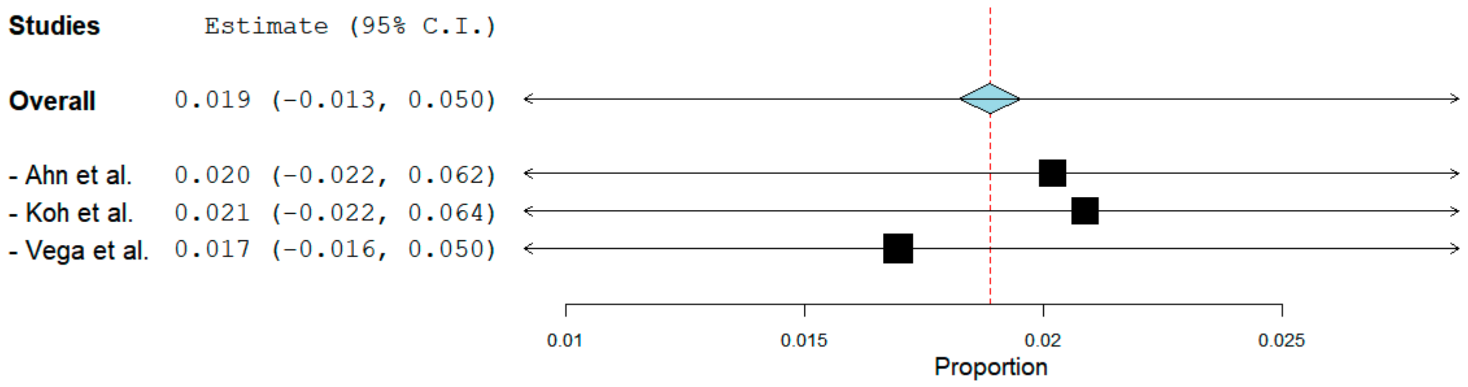

Studies
Estimate (95% C.I.)
Overall
0.019 (-0.013, 0.050)
- Ahn et al.
0.020 (-0.022, 0.062)
- Koh et al.
0.021 (-0.022, 0.064)
- Vega et al.
0.017 (-0.016, 0.050)
0.01
0.015
0.02
0.025
Proportion

F

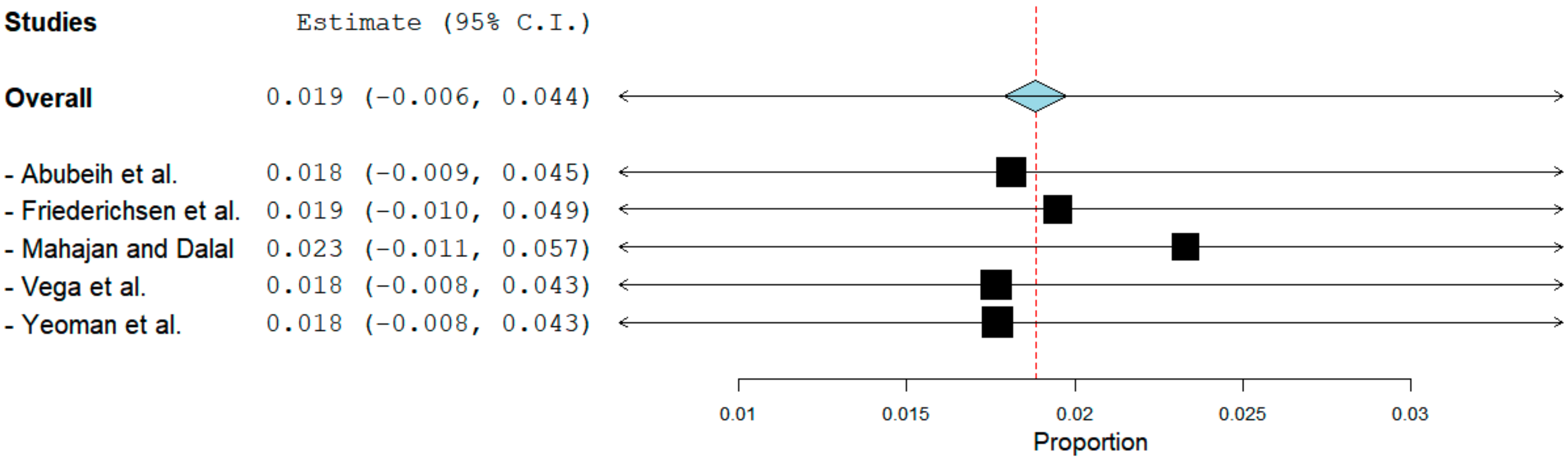
Studies
Estimate (95% C.I.)
Overall
0.019 (-0.006, 0.044)
- Abubeih et al.
0.018 (-0.009, 0.045)
- Friederichsen et al.
0.019 (-0.010, 0.049)
- Mahajan and Dalal
0.023 (-0.011, 0.057)
- Vega et al.
0.018 (-0.008, 0.043)
- Yeoman et al.
0.018 (-0.008, 0.043)
0.01
0.015
0.02
0.025
0.03
Proportion

G

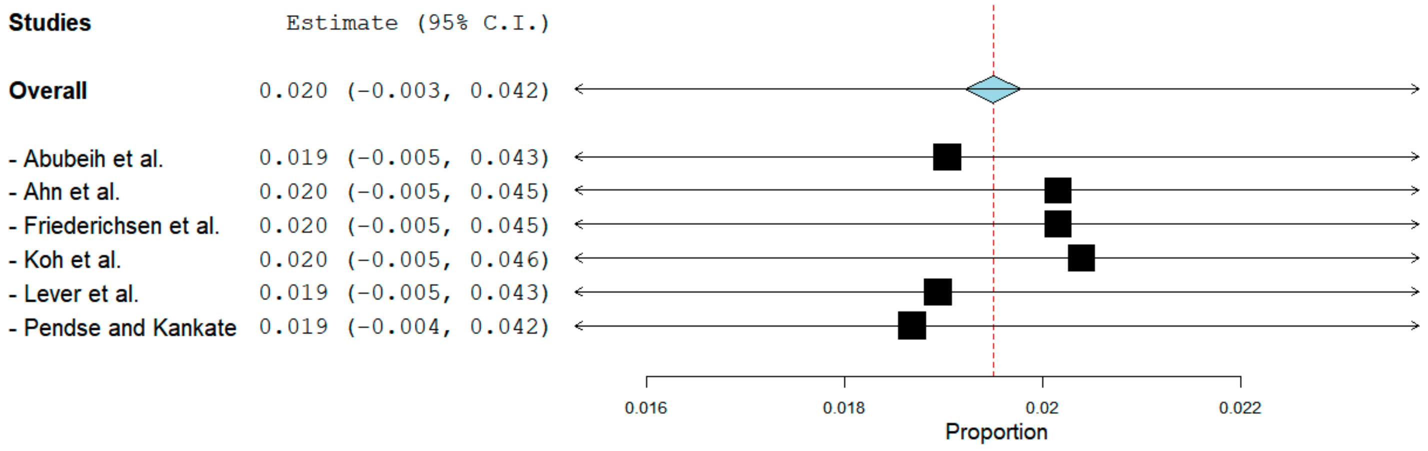

Supplementary Figure S5: Complications leave-one-out analyses: A) Overall, B) Activity limitations, C)Disturbed wound healing, D) Superficial infections, E) Deep infection, F) Nerve injury and G) re-rupture.
