# Supplementary material for: Isolated Flexor Hallucis Longus Tendon Transfer for Chronic Achilles Tendon Rupture: Systematic Review and Meta-Analysis
Source: Healthcare (Basel). 2025 Oct 30;13(21):2751. doi: 10.3390/healthcare13212751 (PMC12607451; doi:10.3390/healthcare13212751)
Supplement: Supplementary file 1 [file healthcare-13-02751-s001.zip › Supplementary item S1 Actual Search strategy.pdf]

Supplementary Material:

Supplementary Item S1: Actual Search Strategy:

Ovid MEDLINE(R) ALL <1946 to July 31, 2025>

1	Achilles tendon rupture*.ti,ab.	2341
2	tendon injuries/ or tendinopathy/	22625
3	Achilles Tendon/	10298
4	calcaneal tendon.ti,ab.	251
5	tendon injur*.ti,ab.	4313
6	hallux.ti,ab.	7175
7	Hallux/	2288
8	Calcaneus/	8146
9	Calcaneus.ti,ab.	6628
10	Tendinopathy.ti,ab.	5923
11	Tendinopathy/	7594
12	insertional Achilles tendinopathy.ti,ab.	302
13	chronic Achilles rupture.ti,ab.	10
14	1 or 2 or 3 or 4 or 5 or 6 or 7 or 8 or 9 or 10 or 11 or 12 or 13	51276
15	((Flexor hallucis longus or FHL) and graft*).ti,ab.	88

16	Tendon transfer.ti,ab.	2525
17	Plastic Surgery Procedures/ or Tendon Transfer/	68315
18	Achilles repair*.ti,ab.	101
19	Suture Techniques/	46633
20	Autografts/	4510
21	Autograft*.ti,ab.	21544
22	Suture Technique*.ti,ab.	3849
23	Plastic Surgery Procedure*.ti,ab.	672
24	FHL augmentation.ti,ab.	8
25	Flexor hallucis longus augmentation.ti,ab.	6
26	surgical flaps*.ti,ab.	167
27	Surgical Flaps/	61871
28	Minimally invasive surgical procedure*.ti,ab.	885
29	Minimally Invasive Surgical Procedures/	32301
30	secondary repair*.ti,ab.	466
31	double bundle.ti,ab.	1421
32	graft*.ti,ab.	414238
33	surgical technique*.ti,ab.	81870

34 15 or 16 or 17 or 18 or 19 or 20 or 21 or 22 or 23 or 24 or 25 or 26 or 27 or 28 or 29 or 30 or 31 or 32 or 33 654639

35 "Range of Motion, Articular"/ 64850

36 Plantarflexion.ti,ab. 3016

37 dorsiflexion.ti,ab. 8588

38 patient reported outcome measure*.ti,ab. 15126

39 Patient Reported Outcome Measures/ 19169

40 Operative Time/ 21486

41 (Operative and (Time or duration)).ti,ab. 115905

42 Postop* complication*.ti,ab. 99931

43 Postoperative Complications/ 428032

44 Functional outcome*.ti,ab. 70709

45 Range of motion.ti,ab. 52119

46 hallux flexion strength.ti,ab. 6

47 (AOFAS or (American Orthop?edic Foot and Ankle Society and ankle-hindfoot score*)).ti,ab. 4718

48 Validated ankle score*.ti,ab. 1

49 (Visual Analogue Scale* or VAS).ti,ab. 92919

50 American Orthop?edic Foot Ankle Score*.ti,ab. 8

51 AOFAS Foot Ankle Score*.ti,ab. 0

52 (Achilles Tendon Rupture Score* or ARTS).ti,ab. 10949

53 ((Foot and Ankle Ability Measure) or FAAM).ti,ab. 628

54 ((Foot and Ankle Disability Index) or FADI).ti,ab. 258

55 patient* satisfaction.ti,ab. 59562

56 Patient Satisfaction/ 95700

57 augmentation.ti,ab. 76482

58 Tendo Achilles.ti,ab. 267

59 (Flexor hallucis longus or FHL).ti,ab. 1908

60 reconstruction.ti,ab. 283872

61 35 or 36 or 37 or 38 or 39 or 40 or 41 or 42 or 43 or 44 or 45 or 46 or 47 or 48 or 49 or 50 or 51 or 52 or 53 or 54 or 55 or 56 943812

62 14 or 58 51377

63 34 or 57 or 59 or 60 913653

64 61 and 62 and 63 3428

65 limit 64 to yr="2024 -Current" **275**

Embase <1974 to 2025 July 30>

1 Achilles tendon rupture*.ti,ab. 2712

2 achilles tendon rupture/ or tendon rupture/ 9474

3 tendon injury/ 10648

4 tendinopathy.ti,ab. 7620

5 achilles tendon/ 13336

6 calcaneal tendon.ti,ab. 319

7 tendon injur*.ti,ab. 5010

8 hallux.ti,ab. 8711

9 hallux/ 5016

10 calcaneus/ 10144

11 Calcaneus.ti,ab. 8131

12 Tendinopathy.ti,ab. 7620

13 tendinitis/ 14465

14 insertional Achilles tendinopathy.ti,ab. 348

15 chronic Achilles rupture.ti,ab. 14

16 1 or 2 or 3 or 4 or 5 or 6 or 7 or 8 or 9 or 10 or 11 or 12 or 13 or 14 or 15 68076

17 ((Flexor hallucis longus or FHL) and graft*).ti,ab. 110

18 Tendon transfer.ti,ab. 2857

19 tendon reconstruction/ or flexor hallucis longus muscle/ 7452

20	Tendon transfer.ti,ab.	2857
21	tendon transfer/	5087
22	plastic surgery/	70220
23	Achilles repair*.ti,ab.	109
24	surgical technique/	437952
25	suture technique/	35334
26	autograft/	19330
27	Autograft*.ti,ab.	26438
28	Suture Technique*.ti,ab.	4718
29	Plastic Surgery Procedure*.ti,ab.	709
30	FHL augmentation.ti,ab.	9
31	Flexor hallucis longus augmentation.ti,ab.	7
32	surgical flaps*.ti,ab.	196
33	surgical flaps/	9524
34	Minimally invasive surgical procedure*.ti,ab.	1238
35	minimally invasive surgery/	63716
36	secondary repair*.ti,ab.	623
37	double bundle.ti,ab.	1710
38	graft*.ti,ab.	570065

39 surgical technique*.ti,ab. 111858

40 augmentation.ti,ab. 96738

41 (Flexor hallucis longus or FHL).ti,ab. 2501

42 reconstruction.ti,ab. 343342

43 17 or 18 or 19 or 20 or 21 or 22 or 23 or 24 or 25 or 26 or 27 or 28 or 29 or 30 or 31 or 32 or 33 or 34 or 35 or 36 or 37 or 38 or 39 or 40 or 41 or 42 1484280

44 "range of motion"/ 83048

45 Plantarflexion.ti,ab. 3696

46 ankle plantarflexion angle/ 490

47 dorsiflexion.ti,ab. 11294

48 ankle dorsiflexion angle/ 796

49 patient reported outcome measure*.ti,ab. 21779

50 patient-reported outcome/ 81965

51 operation duration/ 176762

52 (Operative and (Time or duration)).ti,ab. 218429

53 Postop* complication*.ti,ab. 144185

54 postoperative complication/ 465951

55 Functional outcome*.ti,ab. 103934

56 Range of motion.ti,ab. 67097

57 hallux flexion strength.ti,ab. 10

58 (AOFAS or (American Orthop?edic Foot and Ankle Society and ankle-hindfoot score*)).ti,ab. 5651

59 Validated ankle score*.ti,ab. 1

60 (Visual Analogue Scale* or VAS).ti,ab. 154351

61 visual analog scale/ 159155

62 American Orthop?edic Foot Ankle Score*.ti,ab. 11

63 exp "American Orthopedic Foot and Ankle Society score"/ 2983

64 AOFAS Foot Ankle Score*.ti,ab. 0

65 (Achilles Tendon Rupture Score* or ARTS).ti,ab. 13576

66 ((Foot and Ankle Ability Measure) or FAAM).ti,ab. 819

67 ((Foot and Ankle Disability Index) or FADI).ti,ab. 393

68 patient* satisfaction.ti,ab. 92629

69 patient satisfaction/ 197592

70 44 or 45 or 46 or 47 or 48 or 49 or 50 or 51 or 52 or 53 or 54 or 55 or 56 or 57 or 58 or 59 or 60 or 61 or 62 or 63 or 64 or 65 or 66 or 67 or 68 or 69 1366487

71 16 and 43 and 70 6204

72 limit 71 to yr="2024 -Current" **680**

Search Name:

Date Run: 01/08/2025 19:39:01

Comment:

ID	Search	Hits
#1	((Achilles tendon rupture*)):ti,ab,kw	424
#2	MeSH descriptor: [Tendon Injuries] this term only	415
#3	MeSH descriptor: [Tendinopathy] this term only	909
#4	MeSH descriptor: [Achilles Tendon] explode all trees	474
#5	((calcaneal tendon)):ti,ab,kw	68
#6	((tendon injur*)):ti,ab,kw	2606
#7	((hallux)):ti,ab,kw	818
#8	MeSH descriptor: [Hallux] this term only	34
#9	MeSH descriptor: [Calcaneus] explode all trees	210
#10	((Tendinopathy)):ti,ab,kw	1743
#11	((insertional Achilles tendinopathy)):ti,ab,kw	76
#12	((chronic Achilles rupture)):ti,ab,kw	21

#13	#1 or #2 or #3 or #4 or #5 or #6 or #7 or #8 or #9 or #10 or #11 or #12	5330
#14	(((Flexor hallucis longus or FHL) and graft*)):ti,ab,kw	3
#15	((tendon transfer)):ti,ab,kw	185
#16	MeSH descriptor: [Plastic Surgery Procedures] explode all trees	13607
#17	MeSH descriptor: [Tendon Transfer] explode all trees	78
#18	((Achilles repair*)):ti,ab,kw	213
#19	MeSH descriptor: [Suture Techniques] this term only	2373
#20	((Autograft*)):ti,ab,kw	2277
#21	MeSH descriptor: [Autografts] this term only	303
#22	((Tendo Achilles)):ti,ab,kw	45
#23	((Suture Technique*)):ti,ab,kw	5101
#24	((Plastic Surgery Procedure*)):ti,ab,kw	2247
#25	((FHL augmentation)):ti,ab,kw	2
#26	((Flexor hallucis longus augmentation)):ti,ab,kw	4
#27	((surgical flaps*)):ti,ab,kw	2320
#28	MeSH descriptor: [Surgical Flaps] this term only	1649
#29	((Minimally invasive surgical procedure*)):ti,ab,kw	3759
#30	MeSH descriptor: [Minimally Invasive Surgical Procedures] this term only	1470
#31	((secondary repair*)):ti,ab,kw	6108

#32 ((double bundle)):ti,ab,kw 520

#33 ((graft*)):ti,ab,kw 39688

#34 ((surgical technique*)):ti,ab,kw 31826

#35 #14 or #15 or #16 or #17 or #18 or #19 or #20 or #21 or #22 or #23 or #24 or #25 or #26 or #27 or #28 or #29 or #30 or #31 or #32 or #33 or #34 89997

#36 MeSH descriptor: [Range of Motion, Articular] this term only 7023

#37 ((Plantarflexion)):ti,ab,kw 511

#38 ((dorsiflexion)):ti,ab,kw 2093

#39 ((patient reported outcome measure*)):ti,ab,kw 27712

#40 MeSH descriptor: [Patient Reported Outcome Measures] this term only 2124

#41 MeSH descriptor: [Operative Time] explode all trees 2298

#42 ((Operative and (Time or duration))):ti,ab,kw 33693

#43 ((Postop* complication*)):ti,ab,kw 80585

#44 MeSH descriptor: [Postoperative Complications] explode all trees 56339

#45 ((Functional outcome*)):ti,ab,kw70487

#46 ((Range of motion)):ti,ab,kw 23047

#47 ((hallux flexion strength)):ti,ab,kw 19

#48 (AOFAS or (American Orthop?edic Foot and Ankle Society and ankle-hindfoot score*)):ti,ab,kw 668

#49 (Validated ankle score*):ti,ab,kw 150

#50 ((Visual Analogue Scale* or VAS)):ti,ab,kw 98002

#51 (American Orthop?edic Foot Ankle Score*):ti,ab,kw 496

#52 (AOFAS Foot Ankle Score*):ti,ab,kw 424

#53 ((Achilles Tendon Rupture Score* or ARTS)):ti,ab,kw 1313

#54 ((Foot and Ankle Ability Measure) or FAAM):ti,ab,kw 404

#55 ((Foot and Ankle Disability Index) or FADI):ti,ab,kw 206

#56 ((patient* satisfaction)):ti,ab,kw 65569

#57 MeSH descriptor: [Patient Satisfaction] explode all trees 15772

#58 #36 or #37 or #38 or #39 or #40 or #41 or #42 or #43 or #44 or #45 or #46 or #47 or #48 or #49 or #50 or #51 or #52 or #53 or #54 or #55 or #56 or #57 335592

#59 ((augmentation)):ti,ab,kw 9288

#60 ((Flexor hallucis longus or FHL)):ti,ab,kw 96

#61 ((reconstruction)):ti,ab,kw 11654

#62 #35 or #59 or #60 or #61 103573

#63 #13 and #58 and #62 1182

1 review, 118 trials
